# A quadruple blind, randomised controlled trial of gargling agents in reducing intraoral viral load among hospitalised COVID-19 patients: A structured summary of a study protocol for a randomised controlled trial

**DOI:** 10.1186/s13063-020-04634-2

**Published:** 2020-09-14

**Authors:** Farhan Raza Khan, Syed Murtaza Raza Kazmi, Najeeha Talat Iqbal, Junaid Iqbal, Syed Tariq Ali, Syed Akbar Abbas

**Affiliations:** 1 Department of Surgery, Aga Khan University, Karachi, 74800 Pakistan; 2 Department of Pediatrics & Child Health, Aga Khan University, Karachi, 74800 Pakistan; 3 Department of Chemistry, University of Karachi, Karachi, 75271 Pakistan

**Keywords:** COVID-19, randomised controlled trial, protocol, gargles, nasal lavage, viral load

## Abstract

**Objectives:**

1- To compare the effectiveness of 1% Hydrogen peroxide, 0.2% Povidone-Iodine, 2% hypertonic saline and a novel solution Neem extract (*Azardirachta indica*) in reducing intra-oral viral load in COVID-19 positive patients.

2- To determine the salivary cytokine profiles of IL-2, IL-4, IL-6, IL-10, TNF-α, IFN-γ and IL- 17 among COVID-19 patients subjected to 1% Hydrogen peroxide, 0.2% Povidone-Iodine, 2% hypertonic saline or Neem extract (*Azardirachta indica)* based gargles.

**Trial design:**

This will be a parallel group, quadruple blind-randomised controlled pilot trial with an add on laboratory based study.

**Participants:**

A non-probability, purposive sampling technique will be followed to identify participants for this study.

The clinical trial will be carried out at the Aga Khan University Hospital (AKUH), Karachi, Pakistan. The viral PCR tests will be done at main AKUH clinical laboratories whereas the immunological tests (cytokine analysis) will be done at the Juma research laboratory of AKUH.

The inclusion criteria are laboratory-confirmed COVID-19 positive patients, male or female, in the age range of 18-65 years, with mild to moderate disease, already admitted to the AKUH. Subjects with low Glasgow coma score, with a history of radiotherapy or chemotherapy, who are more than 7 days past the onset of COVID- 19 symptoms, or intubated or edentulous patients will be excluded. Patients who are being treated with any form of oral or parenteral antiviral therapy will be excluded, as well as patients with known pre-existing chronic mucosal lesions such as lichen planus.

**Intervention and comparator:**

Group A (n=10) patients on 10 ml gargle and nasal lavage using 0.2% Povidone-Iodine (Betadiene® by Aviro Health Inc./ Pyodine® by Brooks Pharma Inc.) for 20-30 seconds, thrice daily for 6 days. Group B (n=10) patients will be subjected to 10 ml gargle and nasal lavage using 1% Hydrogen peroxide (HP® by Karachi Chemicals Products Inc./ ActiveOxy® by Boumatic Inc.) for 20-30 seconds, thrice daily for 6 days. Group C will comprised of (n=10) subjects on 10ml gargle and nasal lavage using Neem extract solution (*Azardirachta indica*) formulated by Karachi University (chemistry department laboratories) for 20-30 seconds, thrice daily for 6 days. Group D (n=10) patients will use 2% hypertonic saline (Plabottle® by Otsuka Inc.) gargle and nasal lavage for a similar time period. Group E (n=10) will serve as positive controls. These will be given simple distilled water gargles and nasal lavage for 20-30 seconds, thrice daily for six days. For nasal lavage, a special douche syringe will be provided to each participant. Its use will be thoroughly explained by the data collection officer. After each use, the patient is asked not to eat, drink, or rinse their mouth for the next 30 minutes.

**Main outcomes:**

The primary outcome is the reduction in the intra-oral viral load confirmed with real time quantitative PCR.

**Randomisation:**

The assignment to the study group/ allocation will be done using the sealed envelope method under the supervision of Clinical Trial Unit (CTU) of Aga Khan University, Karachi, Pakistan. The patients will be randomised to their respective study group (1:1:1:1:1 allocation ratio) immediately after the eligibility assessment and consent administration is done.

**Blinding (masking):**

The study will be quadruple-blinded. Patients, intervention provider, outcome assessor and the data collection officer will be blinded. The groups will be labelled as A, B, C, D or E. The codes of the intervention will be kept in lock & key at the CTU and will only be revealed at the end of study or if the study is terminated prematurely.

**Numbers to be randomised (sample size):**

As there is no prior work on this research question, so no assumptions for the sample size calculation could be made. The present study will serve as a pilot trial. We intend to study **50** patients in five study groups with 10 patients in each study group. For details, please refer to Fig. 1 for details.

**Trial Status:**

Protocol version is 7.0, approved by the department and institutional ethics committees and clinical trial unit of the university hospital. Recruitment is planned to start as soon as the funding is sanctioned. The total duration of the study is expected to be 6 months i.e. August 2020-January 2021.

**Trial registration:**

This study protocol was registered at www.clinicaltrials.gov on 10 April 2020 NCT04341688.

**Full protocol:**

The full protocol is attached as an additional file, accessible from the Trials website (Additional file 1). In the interest in expediting dissemination of this material, the familiar formatting has been eliminated; this Letter serves as a summary of the key elements of the full protocol. The study protocol has been reported in accordance with the Standard Protocol Items: Recommendations for Clinical Interventional Trials (SPIRIT) guidelines (Additional file 2).

**Fig. 1:**
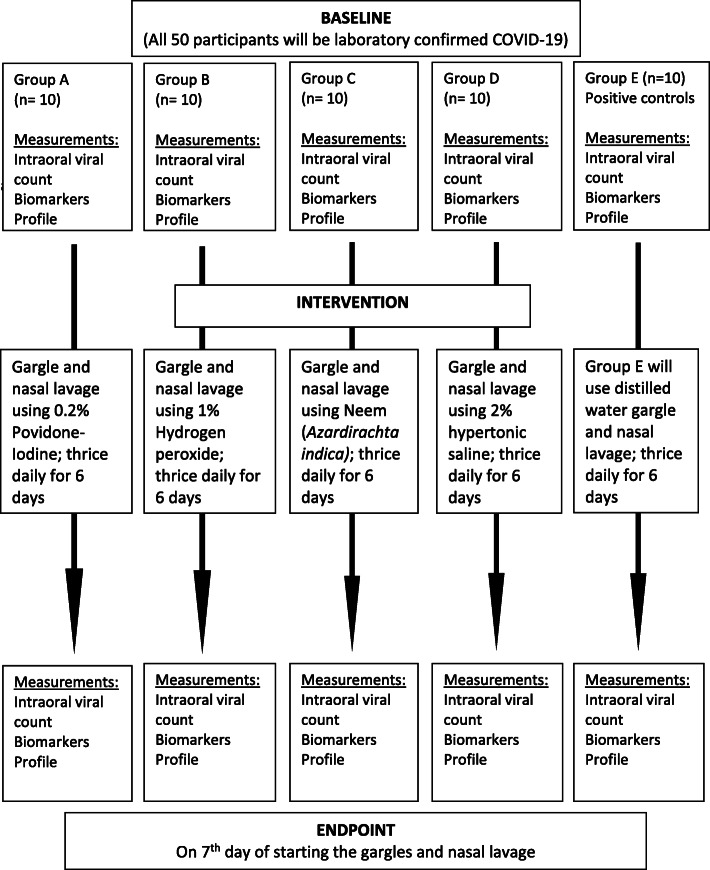
Flow diagram of study-participants’ timeline

## Supplementary information

**Additional file 1.** Full Study Protocol.

**Additional file 2.** SPIRIT 2013 Checklist: Recommended items to address in a clinical trial protocol and related documents.

## Data Availability

Individual patient data will be kept confidential and no account personal identifiers of the study participants disclosed to the public. Only the study investigators and clinical trial unit (CTU) of the Aga Khan University, Karachi, Pakistan will have access to the study data. CTU reserves the right of carrying out study audit by internal or external auditors anytime.

