# Supplementary material for: A quadruple blind, randomised controlled trial of gargling agents in reducing intraoral viral load among hospitalised COVID-19 patients: A structured summary of a study protocol for a randomised controlled trial
Source: Trials. 2020 Sep 14;21:785. doi: 10.1186/s13063-020-04634-2 (PMC7487448; doi:10.1186/s13063-020-04634-2)
Supplement: Supplementary file 1 — Additional file 1. Full Study Protocol. [file 13063_2020_4634_MOESM1_ESM.pdf]

A double blind, randomized controlled pilot trial of gargling agents in reducing intraoral viral load among laboratory confirmed COVID-19 patients: GARGLES STUDY {1}

Study protocol registered at www.clinicaltrials.gov with # NCT 04341688 {2a and 2b}

Protocol version 7.0 dated 21-06-2020 {3}

Farhan Raza Khan, BDS, MS, MCPS, FCPS
Associate Professor, Operative Dentistry
Department of Surgery, Aga Khan University
farhan.raza@aku.edu
ORCID# 0000-0002-5650-6268

Syed Murtaza Raza Kazmi, BDS, FCPS
Assistant Professor, Prosthodontics
Department of Surgery, Aga Khan University
syed.murtaza@aku.edu
ORCID: 0000-0002-6934-6945

Najeeha Talat Iqbal, PhD
Associate Professor, Research
Department of Pediatrics & Child Health, Aga Khan University
najeeha.iqbal@aku.edu

Junaid Iqbal, PhD
Assistant Professor, Research,
Department of Pediatrics & Child Health, Aga Khan University
junaid.iqbal@aku.edu

Syed Tariq Ali, PhD
Associate Professor, Chemistry
Department of Chemistry, University of Karachi
stali@uok.edu.pk

Syed Akbar Abbas, MBBS, FCPS
Assistant Professor, Otolaryngology
Department of Surgery, Aga Khan University
akbar.abbas@aku.edu
ORCID: 0000-0003-3911-1632

Funding Body/ Sponsor {4}: To be identified.

Roles & Responsibilities of the investigators {5a}:

**Investigators**	**Roles & Responsibilities**
**Farhan Raza Khan**	Concept, methodology, critical review and overall supervision of the study. Responsible for reporting protocol amendments.
**Syed Murtaza Raza Kazmi**	Literature review, protocol writing, coordination with CTU and supervision of clinical data collection.
**Najeeha Talat Iqbal**	Will oversee the cytokine work and immunological phase of the study.
**Junaid Iqbal**	Will oversee the virology part of the study, Real-time PCR work etc.
**Syed Tariq Ali**	Compounding the gargling agent from *Azardirachta indica* and overseeing the chemistry part of the trial.
**Syed Akbar Abbas**	Will oversee the clinical part of the study, especially specimen collection from oro-naso-pharynx.
**Data collection officer** (to be appointed)	Collect the samples from the COVID-19 positive patients and sending it to the Juma laboratory.

Trail sponsor {5b): To be identified.

Role of study sponsor and funders {5c}: The funds from the granting will be used in procuring the COVID-19 kits, CBA biomarker kits and laboratory consumables etc. Funders will have no role in study design; data collection, management, analysis, and interpretation of data and writing or dissemination of the final report**.**

Trial Coordination Center {5d}: Aga Khan University Hospital, Karachi, Pakistan. The Clinical Trial Unit (CTU) of Aga Khan University, Karachi, Pakistan will serve as the trial overseeing committee.

A quadruple blind, randomized controlled pilot trial of gargling agents in reducing intraoral viral load among laboratory confirmed COVID-19 patients: GARGLES STUDY

INTRODUCTION: {6a}

The outbreak of coronavirus disease originated in Wuhan City, in the late December 2019 has been declared as a pandemic by World Health Organization (WHO) in March 2020. (1) Coronavirus is an RNA enveloped virus that is closely related to SARS-Cov-2 family. The present pandemic has affected almost two-hundred countries of the world. As of June 01, 2020, there are at least 6,281,750 individuals affected with COVID-19 infection, globally. Of these 2,854,299 (45.4%) have already recovered and 374,229 (5.9%) have died. The status of presently infected 3,427,451 individuals reveals that 3,000,107 (98%) are likely to recover owing to their mild condition but the other 53,398 (2%) are in critical condition and have significantly high risk of dying. (1) In Pakistan, to date over 72,460 people have been affected with this virus, 1,543 deaths recorded and another 100 patients are present in critical condition. (2)

COVID-19 can be transmitted directly or indirectly through saliva, aerosol, micro-droplets and eye secretions. (3) Oral cavity acts as an area for the dissemination of pathogens to far-off body parts, (4) especially among the patients where immunity is compromised. An antimicrobial mouth rinse is generally believed to reduce the number of oral microbes including viruses. Dentists, dental hygienists and para-dental auxiliaries are at considerably higher risk of acquiring and transmitting COVID-19 infection owing to routine use of aerosol generating procedures in dental profession. In the context of coronavirus disease outbreak, the American Dental Association (ADA) and International Association of Dental Research (IADR) have globally recommended the dentists (5) to stop all the elective dental procedures (scalings, crowns, orthodontics, and implants etc.) and just limit their work to emergency work such as management of dental pain, bleeding, swelling and dento-facial trauma. This has globally

affected the dental care of patients. Moreover, the impact of halting the dental care on the economics of the profession is beyond calculation.

Chlorhexidine is a widely used mouth rinse in dental practice. (6) Its a cationic bisguanide with broad spectrum antimicrobial activity. However, it may not be effective against COVID-19. Moreover, its ototoxicity and association with exacerbation of respiratory tract infections (7, 8) precludes its use in potential coronavirus disease patients. Recently, ADA has recommended a pre-procedural mouth rinsing and gargle with 1% hydrogen peroxide. Other investigators have documented the efficacy of 0.2% Povidone-Iodine (9) in reducing the intra-oral viral load of corona viruses such as SARS-CoV and MERS CoV.

In a recent study done on critically ill COVID-19 patients in Wuhan, China, Huang *et al.* suggested that there is a cytokine storm (i.e. higher levels of IL2, IL7, IL10, GSCF, IP10, MCP1, MIP1A, and TNFα) which could be related to the severity of disease. (10) Another study on COVID-19 participants in China, demonstrated that increased expression of IL2 and IL6 in serum is correlated with the severity and prognosis disease. (11) Moreover, the biopsy of subjects died of coronavirus disease showed lymphocytes infiltrating the lungs interstitium with cytometric analysis showing significant over-activation of T cells. (12) Thus, it's imperative to study the cytokine profile in COVID-19 patients.

Rationale: {6b}

There is a need to identify a gargling agent that has maximum efficacy against COVID-19 so that it could be used as a preoperative gargle especially before embarking upon clinical examination of (or initiating any dental procedures in) symptomatic (and more importantly asymptomatic) COVID-19 patients. Moreover, we intend to explore the efficacy of a novel compound for gargle (derived from extracts of Neem tree) in reducing intra-oral viral load in COVID-19 positive patients. Neem leaf (*Azardirachta indica*) has a number of active chemicals that have antiviral activity against a number of viruses. (13, 14) Being an anti-inflammatory compound, Neem based gargles and / or nasal douche are expected to reduce the colonies of COVID-19 in mouth and oro-pharynx, without causing any significant adverse effects.

Hypertonic saline can be considered as a potential intervention for reducing viral load in the oro-naso-pharynx. Given the apparent mechanical rather than blood-borne slow advance of coronavirus from oro-naso-pharnyx to lung, the possibility exists that reducing viral load through debriding could aid effective immune response in the same way that debriding burns reduces time of healing. Hypertonic saline rinses have had variable results. At least one RCT (done recently in Edinburgh, UK) showed statistically significant result in upper respiratory tract infection. (15) Investigators used hypertonic saline nasal irrigation and gargles as an intervention and observed that the duration of illness was lowered by 1.9 days ($p = 0.01$), over-the-counter medications use by 36% ($p = 0.004$), transmission within household contacts by 35% ($p=0.006$) and viral shedding by $\geq 0.5 \log^{10}$/day ($p = 0.01$). (15) Data from a Pakistan based study also suggests that nebulization with hypertonic saline yield favorable outcomes in management of viral respiratory tract infection compared to normal saline. (16)

Primary Objective: {7a}

- To compare the effectiveness of 1% Hydrogen peroxide, 0.2% Povidone-Iodine, 2% hypertonic saline and a novel solution Neem extract (*Azardirachta indica*) in reducing intra-oral viral load in COVID-19 positive patients.

Secondary Objective: {7b}

- To determine the salivary cytokine profiles of IL-2, IL-4, IL-6, IL-10, TNF-α, IFN-γ and IL-17 among COVID-19 patients subjected to 1% Hydrogen peroxide, 0.2% Povidone-Iodine, 2% hypertonic saline or Neem extract (*Azardirachta indica)* based gargles.

Hypotheses:

- 0.2% Povidone-Iodine gargle has a higher antiviral efficacy against COVID-19 than 1% Hydrogen peroxide gargle or 2% hypertonic saline gargle.
- Neem extract (*Azardirachta indica)* can be used as an antiviral gargle in COVID-19 positive patients (similar to established gargling agents such as 1% Hydrogen peroxide or 0.2% Povidone-Iodine).

MATERIALS & METHODS:

Trial design {8}: It will be a parallel group, quadruple blind-randomized controlled pilot trial annexed with a laboratory based study.

Study settings & duration {9}: The clinical trial will be carried out at the Aga Khan University Hospital (AKUH), Karachi, Pakistan. The total duration of the study is expected to be 6 months i.e. August 2020-January 2021. The viral PCR tests will be done at main AKUH clinical laboratories whereas the immunological tests (cytokine analysis) will be done at the Juma research laboratory of AKUH. The study protocol is already registered at www.clinicaltrials.gov with ID# NCT 04341688.

Sampling technique & Eligibility criteria {10}: Non probability, purposive sampling technique will be followed to identify a total of 50 participants for this study. The inclusion criteria are laboratory confirmed COVID-19 positive males or females (in the age range of 18-65 years), with mild to moderate disease, already admitted in the AKUH. Subjects who have more than 7 days of the onset of COVID-19 symptoms, edentulous patients, subjects with low Glasgow coma score, intubated patients, history of radiotherapy or chemotherapy will be excluded. Patients who are being treated with any form of oral or parenteral antiviral therapy will be excluded. Patients with known pre-existing chronic mucosal lesions such as lichen planus will also be excluded. Patients who are allergic to the study drugs (Povidone-Iodine, Hydrogen Peroxide, Neem Extract or Hypertonic Saline) will also be excluded.

Intervention {11a}: Group A (n=10) patients on 10 ml gargle and nasal lavage using 0.2% Povidone-Iodine (Betadiene® by Aviro Health Inc./ Pyodine® by Brooks Pharma Inc.) for 20-30 seconds, thrice daily for 6 days. Group B (n=10) patients will be subjected to 10 ml gargle and nasal lavage using 1% Hydrogen peroxide (HP® by Karachi Chemicals Products Inc./ ActiveOxy® by Boumatic Inc.) for 20-30 seconds, thrice daily for 6 days. Group C will comprised of (n=10) subjects on 10ml gargle and nasal lavage using Neem extract solution (*Azardirachta indica*)

formulated by Karachi University (chemistry department laboratories) for 20-30 seconds, thrice daily for 6 days. Group D (n=10) patients will use 2% hypertonic saline (Plabottle® by Otsuka Inc.) gargle and nasal lavage for a similar time period. Group E (n=10) will serve as positive controls. These will be given simple distilled water gargles and nasal lavage for 20-30 seconds, thrice daily for six days. For nasal lavage, a special douche syringe will be provided to each participant. Its use will be thoroughly explained by the data collection officer. After each use, patient is not supposed to eat, drink or mouth rinse for next 30 minutes.

Discontinuation of intervention {11b}: Patients who develop rashes, or any other mucosal reaction in oral mucosa or patients who do not wish to continue with the gargles and nasal lavage will be discontinued in the study.

Compliance on intervention {11c}: The data collection officer will monitor the amount of gargling solution consumed on daily basis to record the patient compliance on intervention. And once the study is over, the residual bottle will be collected and sent to the incineration. Similarly, any unused/ partially used study drug bottle will be destroyed via incineration.

Concomitant care {11d}: Study participants will continue to get their needed routine inpatient medical/pulmonology care concomitantly as per the individual requirements under the care of the attending physician(s).

Outcomes {12}: Primary outcome is the reduction in the intra-oral viral load confirmed with real time PCR. Secondary outcome is the change in inflammatory biomarkers (IL-2, IL-4, IL-6, IL-10, TNF-α, IFN-γ and IL-17) profiles in the five study groups.

Participants' timeline {13}: The timeline has been added as a flow diagram at the end of the protocol. Please see figure 1 on page no. 15.

Sample Size {14}: As there is no prior work on this research question, so no assumptions for the sample size calculation could be made. The present study will serve as a pilot trial. We intend to study **50** patients in five study groups. For details, please refer to figure 1 on page no. 15.

Recruitment {15}: Patients who are laboratory confirmed COVID-19 within 7 days of the onset of their symptoms who are already admitted as inpatient at the Aga Khan University Hospital, Karachi, Pakistan will be contacted for a voluntarily participation in the study.

Group allocation/ Randomization {16a}: The assignment to the study group/ allocation will be done using sealed envelope method under the supervision of Clinical Trial Unit (CTU) of Aga Khan University, Karachi, Pakistan. The patients will be randomized to their respective study group immediately after the eligibility assessment and consent administration is done.

Allocation concealment {16b}: All the study drugs will be kept in identical dark colored bottles so that the intervention is masked.

Implementation {16c}: The Neem extract (*Azadirachta indica*) will be compounded at the Chemistry department, University of Karachi, Pakistan. This will be arranged from there whereas other drugs are stored at AKUH pharmacy/ distribution department. The CTU will ensure that only labels mentioning A, B, C, D or E are pasted on the bottles.

Blinding/ masking {17a}: Study will be blinded in quadruple. Patients, intervention provider, outcome assessor and the data collection officer will be blinded. The groups will be labelled as A, B, C, D or E. The codes of the intervention will be kept in lock & key at the CTU and will only be revealed at the end of study or if the study is terminated prematurely.

Un-blinding {17b}: Although, un-blinding is not permissible, however, if a participant recognizes the intervention using his/her prior knowledge or experience; they will still be encouraged to continue with the study drug till the end of the study. But if any patients develops a serious reaction to the intervention drug, then un-blinding will be done. The principal investigator will consult CTU and with their permission and taking primary attending physician onboard, un-blinding for the patient in question will be done. This will be documented on the proforma.

Specimen collection {18a}: The baseline oral swab will obtained (by a trained data collection officer using the required PPEs) from the posterior pharyngeal wall/ tonsillar area of the study participant on day one before initiating the gargles. Study participants will continue to use the

gargle and nasal lavage for 6 days. The end-point oral-pharyngeal swab will be taken on the day 7. Participants will be provided with a protective hood so that they themselves don't generate aerosols in the immediate vicinity while carrying out the gargling and nasal lavage. For nasal lavage, they will use the special douche syringe as described earlier.

Retention of study participants {18b}: To retain maximum number of patients in the study, the data collection officer will keep the contact number of all the recruited patients, so that even if they are discharged from the hospital, they will be encouraged to continue with the study protocol and present to us on the day 7 at the AKUH dedicated COVID-19 testing facility for the collection of their endpoint pharyngeal swabs.

Data management {19}: A trained data collection officer will be responsible for data entry, coding, security, and storage. To ensure data quality, double data entry will be followed. The data collection officer will ensure coordination with the investigators and the statistician.

The period of retention of the data will be according to the CTU policy on this subject i.e. 15 years after the completion of the trial. The biological samples (pharyngeal swabs), and the partially consumed study drug will be discarded via incineration immediately after the data collection.

Data Analysis {20}: Data will be analyzed using SPSS for Windows (version 23.0 SPSS) and Graph Pad Prism 7.0 software.
Mean and standard deviation of the continuous variables (age, COVID-19 viral counts and cytokine profiles at baseline and endpoint etc.) will be computed. Frequency distribution of the categorical variables will be determined (gender, co-morbids, presence of hypertension, diabetes, periodontal status etc.) Repeated measures ANOVA will be used to compare the reduction in intra-oral viral load and the changes in the inflammatory biomarkers in the study groups. In case of substantially low count of participants in the study groups, non-parametric tests such as Kruskal-Wallis or Friedman test will be employed. Generalized Estimation Equation (GEE) will done to predict changes in the COVID-19 viral load and cytokine profiles. A subset analysis using Zero inflated negative binomial (ZINB) model will be employed, provided a

significant reduction in viral load is observed in any of the study groups. A p-value of <0.05 will be taken as statistically significant. {20a}

The **biases and confounders** can be taken care at following four levels:

At recruitment stage, we will use restriction i.e. only those COVID-19 positive subjects will be recruited who have no other major co-morbidities. At intervention assignment, the random group allocation will ensure even distribution of the confounders in the study groups, thus their differential effect on the outcome will be taken care of. At analysis stage, we will employ GEE regression analysis (as described above) to account for biases and confounders and lastly, the subset analysis will stratify the data and nullify the effect of biases.

Interim analysis {20b} is not planned on account of a short duration of the study. Intention to treat analysis (ITT) will be adopted for missing data and protocol violations or deviations by uncompliant patients. {20c}

Data monitoring {21a}: It will be done bimonthly at two levels. The data monitoring committee comprising of principal and co-investigators will be responsible for the monitoring the data at the primary level whereas the CTU at the university level will oversee and monitor the overall conduct of the study.

The decision of termination of the study {21b} will be made in consultation with the director CTU and the sponsors/ funding agency. The decision will be made after mutual consensus of the parties.

Harms/ adverse reaction reporting {22}: The adverse events will be documented for each patient and will be reported bimonthly to the clinical trial unit (CTU). This will also documented in the final report. Sponsor/ funders will be notified of the adverse event using their case report. Due to topical nature of the intervention, most likely harm would be an allergic reaction to any of the study drugs [Povidone-Iodine, Hydrogen Peroxide or Neem Extract (*Azadirachta indica*)]. In case of any serious harm, the advice of the primary attending physician will take precedence over others. However, such patient will be discontinued in the trial, CTU and sponsors informed and un-blinding will be followed as prescribed in section 17b. The role of patient’s medical insurance will be decided on individual basis

Study Audit {23}: CTU reserves the right of carrying out study audit by internal or external auditors anytime.

Ethical approval {24}: The study would be initiated after the approval from the institutional and regulatory authorities. The application is being submitted at university ethics review committee (ERC). Provisional approval of the protocol from CTU has already been obtained. Time required to complete the entire study will be around 6 months.

Protocol amendments {25}: One of the investigators (FRK) will be responsible for documentation of the protocol amendments or deviations. These will be conveyed to the other co- investigators, ERC, CTU and the Funders by the aforementioned responsible person.

Consent for the trial and biological specimen testing {26}: Study details will be explained to the participants in local language i.e. Urdu or English for those who are more familiar with the latter by a trained data collection officer {26a} and a written informed consent will be obtained. In addition to the clinical trial, this consent will also entail the use of biological specimens for the laboratory testing and analysis. {26b}

Confidentiality {27}: Individual patient data will be kept confidential and no account personal identifiers of the study participants disclosed to public.

Declaration of Interests {28}: All authors FRK, SMRK, NTI, JI, STI and SAA confirm that they don't have any conflict of interest with the products involved in the study protocol. Similarly, there is no arrangement that could constitute a conflict of interest in any form.

Access to data {29}: Only study investigators and CTU or its auditing team will have the access to the study data.

Ancillary and post-trial care {30}: The two investigators (FRK and SMRK) take up the responsibility of providing ancillary essential dental care to the study participant for six months post trial period.

Dissemination policy {31a}: The trial protocol is already registered at www.clinicaltrials.gov; NCT 04341688. The knowledge generated in this study will be published in peer-reviewed journals of

dentistry and/ or medicine without disclosing any individual patient data. The trial investigators will be the authors in the final report and publication resulting from the study. {31b}.

Informed consent {32}: Informed consent will be obtained in Urdu (or in English for those who are well versed with the latter) language where the harms and benefits of the gargles and nasal lavage will be explained along with the method of their use in a simple language. The details are stated in the Urdu and English consent forms. The signed consent forms will be retained for the record purpose.

Laboratory phase for Biological specimen {33}: Swabs taken from the posterior pharyngeal wall and tonsillar area will be delivered to the laboratory in the transport medium within 4 hours of the specimen collection. The RNA will be extracted in the biosafety cabinet in the BSL-3 laboratory using QIAamp Viral RNA Mini Kit (Qiagen Inc.). Viral RNA will be used as a template in PCR containing following two sets of CDC approved primers and probes targeting COVID-19 virus and Fast Virus 1-Step Master Mix (Thermofisher Inc.). 2019-nCoV_Nl-F (GACCCCAAAATCAGCGAAAT), 2019-nCoV_Nl-R (TCTGGTTACTGCCAGTTGA ATCTG), 2019-nCoV_Nl-P (FAM-ACCCCGCATTACGTTTGGTGGACC-BHQ1) 2019-nCoV_N2-F (TTACAAACATTGGCCGCAAA) 2019-nCoV_N2-R (GCGCGACATTCCGAAGAA), 2019-nCoV_N2-P FAM-(ACAATTTGCCCCCAGCGCTTCAG-BHQ1). To generate standard curve, we will design a synthetic oligo which will contain target sites for the aforementioned forward & reverse primers and probes. We will clone this synthetic oilgo in pGEMT vector after amplifying it through PCR; calculate its copy numbers and use its different dilutions to generate the standard-curve in PCR. Sample Ct values below 35 cycles will be considered as positive. The unknown sample's Ct values will be plotted on standard curve to know the copy numbers.

Cytokine analyses from the collected specimen will be carried out using Cytometric Bead Array (BD Biosciences) on FACS Celesta analyzer. Cytokine bead array detects cytokines using fluorescence labelled beads coated with antibodies. Following incubation of samples with cytokines, a fluorescence labelled antibody is used for the signal detection. The Median Fluorescence Intensity (MFI) is proportional to the amount of analyte present in the sample. All

procedure will be carried as per manufacturer's instructions. Unknown concentration of cytokines will be quantified through a known standards curve of cytokines. Flow JO software will be used for the final analyses.

The cost of the PCR and Cytokine analysis will be borne by the study grant. There will be no financial burden on the patients for any investigation.

Safety and effectiveness of the Neem leaf extract (*Azardirachta indica*) {34]:

There is a substantial evidence on the effectiveness and safety of the Neem extract. Clinical trials have shown that Neem based mouthwashes are effective against chronic gingivitis (17,18) plaque retention (18, 19) and dental caries (20). The antiviral activity of Neem leaf (*Azardirachta indica*) has been established (21) especially herpes simplex virus type-1 (22), group B coxsackie viruses (23) and Novel H1N1 Flu virus. (24)

Regarding extraction process of Neem, (17) it will be done at the Chemistry department laboratories at the University of Karachi, Pakistan. Neem leaves will be dried under controlled condition, then its 20.0 g dry powder will be mixed with 100 ml of 70% (w/v) ethanol for a week in a round bottom flask with occasional shaking. This will be kept in the dark container to avoid effect of light. The extract will then be filtered through filter paper, measured and kept in an airtight container. The final mouthwash formulation to have 25% of Neem extract, 20.0% of Saccharin and Peppermint oil (<0.1%) as flavor. HPLC based analysis will ensure the quality control of the solution. (25)

Why this study is important and why are we doing it?

Pakistan is a resource restraint country, it's not possible to carry out coronavirus testing at mass scale. Simple, cost effective intervention against the present pandemic is highly desirable.

This research is important as there is no definitive management for Covid patients. At present, only preventive strategies such as frequent hand washing, use of mask, social distancing and avoidance of congregation is being followed. We have suggested a RCT to determine

effectiveness of simple mouthwash and nasal lavage (with antiviral agents) as a prophylaxis against viral colonization.

If we are able to demonstrate effectiveness of topical anti-viral mouthwash and nasal lavage then not only dentists, other clinicians involved in aerosol generating procedures (ENT, maxillofacial and anesthesiologists) could be benefited by simply doing a pre-procedure mouthwash and nasal lavage as a prophylaxis before embarking upon an aerosol generating procedure. Moreover, such Gargles based prophylaxis can be part of the routine management of patients visiting outpatient clinics where the status of Covid-19 is not known.

The risk of morbidity and mortality is high among physicians and nurses involved in the screening and management of Covid-19 patients. Globally, over 230 physicians and surgeons have died while taking care of these patients. The cause of death was high exposure of viral load. The antiviral gargles and nasal lavage can decrease the fatalities among doctors and nurses.

The present study will be the first to demonstrate that topical use of antiviral rinses could quantitatively reduce COVID-19 virus in the oro-naso-pharynx. Patients, physicians, nurses and dentists, all could be benefited with this findings of this study. Above all, if this is found to be effective, such gargles could be used as routine prophylaxis of patients visiting outpatient clinics where the status of COVID-19 is not known.

FIGURE 1: FLOW DIAGRAM OF STUDY –PARTICIPANTS' TIMELINE {13}

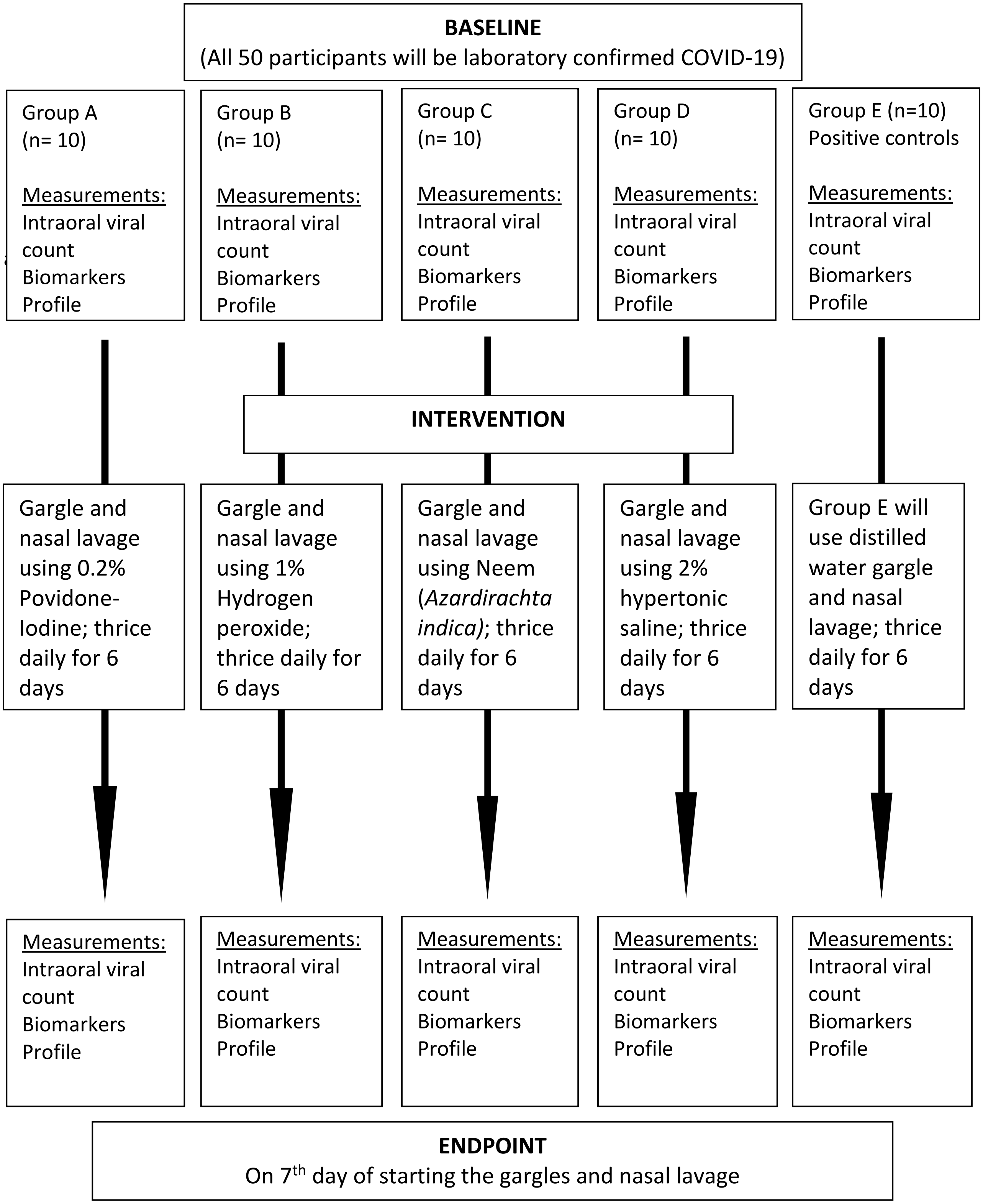

References:

1. https://www.worldometers.info/coronavirus/[Accessed 01 June, 2020].
2. https://www.covid.gov.pk/[Accessed 01 June, 2020].
3. Peng X, Xu X, Li Y, Cheng L, ZhouX, Ren B. Transmission routes of 2019-nCoV and controls in dental practice. Int J Oral Sci. 2020; 12(9):1-6. PMID: 32127517.
4. Shafiq HB, Amin U, Nawaz S. Comparative analysis of various antimicrobial agents present in locally available mouthwashes against oral pathogens. Pak J Pharm Sci. 2018: 31(5):1881-7. PMID: 30150184.
5. Burger D. ADA recommending dentists postpone elective procedures. Available from: https://www.ada.org/en/publications/ada-news/2020-archive/march/ada-recommending-dentists-postpone-elective-procedures[Accessed 5 May, 2020].
6. Tanzer JM, Slee AM, Kamay BA. Structural requirements of guanide, biguanide, and bisbiguanide agents for antiplaque activity. Antimicrob Agents Chemother. 1977;12(6): 721–9. PMID: 931371.
7. Lai P, Coulson C, Pothier DD, Rutka, J. Chlorhexidine ototoxicity in ear surgery, part 1: Review of the literature". J Otolaryngol Head Neck Surg. 2011;40 (6): 437–40. PMID: 22420428.
8. Hirata K, Kurokawa A. Chlorhexidine gluconate ingestion resulting in fatal respiratory distress syndrome. Vet Human Toxicol. 2002; 44 (2): 89–91. PMID: 11931511.
9. Eggers M, Koburger-Janssen T, Eickmann M, Zorn J. In vitro bactericidal and virucidal efficacy of povidone-iodine gargle/mouthwash against respiratory and oral tract pathogens. Infect Dis Ther. 2018; 7(2):249-59. PMID: 29633177.
10. Huang C, Wang Y, Li X, Ren L, Zhao J, Hu Y, *et al.* Clinical features of patients infected with 2019 novel coronavirus in Wuhan, China. Lancet 2020; 395(10223): 497–506. PMID: 31986264.
11. Chen L, Liu HG, Liu W, Liu J, Liu K, Shang J, *et al.* [Analysis of clinical features of 29 patients with 2019 novel coronavirus pneumonia]. Zhonghua Jie He He Hu Xi Za Zhi 2020; 43(0): E005. PMID: 32026671.
12. Xu Z, Shi L, Wang Y, Zhang J, Huang L, Zhang C, *et al.* Pathological findings of COVID-19 associated with acute respiratory distress syndrome. Lancet Respir Med 2020; 8(4): 420-2. PMID: 32085846.
13. Tiwari V, Nissar A. Darmani NA, Beatrice YJ, Yue T, Shukla D. In vitro antiviral activity of neem (Azardirachta indica L.) bark extract against herpes simplex virus type-1 infection. Phytother Res. 2010; 24(8): 1132-40. PMID: 20041417.
14. Ahmad A, Javed MR, Rao AQ, Husnain T. Designing and screening of universal drug from neem (Azadirachta indica) and standard drug chemicals against influenza virus nucleoprotein. BMC Comp Alt Med. 2016; 16:519. PMID: 27986088.
15. Ramalingam S, Graham C, Dove J, Morrice L, Sheikh A. A pilot, open labelled, randomised controlled trial of hypertonic saline nasal irrigation and gargling for the common cold. Sci Rep. 2019; 9: 1015. PMID: 30705369.
16. Salman MK, Ashraf MS, Ahmad E. Comparison of 3.0% hypertonic saline versus 0.9% normal saline nebulization for acute bronchiolitis in children. J Postgrad Med Inst. 2018; 32(2): 128-31.
17. Botelho MA, dos Santos RA, Martins JG, Carvalho CO, Paz MC, Azenha C, Ruela RS, Queiroz DB, Ruela WS, Marinho G, Ruela FI. Efficacy of a mouthrinse based on leaves of the neem tree (Azadirachta indica) in the treatment of patients with chronic gingivitis: A double-blind, randomized, controlled trial. J Med Plant Res. 2008; 2(11): 341-6.

18. Sharma R, Hebbal M, Ankola AV, Murugaboopathy V, Shetty SJ. Effect of two herbal mouthwashes on gingival health of school children. J Tradit Complement Med. 2014; 4(4):272-8. PMID: 25379471.
19. Chatterjee A, Saluja M, Singh N, Kandwal A. To evaluate the antigingivitis and antipalque effect of an Azadirachta indica (neem) mouthrinse on plaque induced gingivitis: A double-blind, randomized, controlled trial. J Indian Soc Periodontol. 2011; 15(4):398-401. PMID: 22368367.
20. Chava VR, Manjunath SM, Rajanikanth AV, Sridevi N. The efficacy of neem extract on four microorganisms responsible for causing dental caries viz Streptococcus mutans, Streptococcus salivarius, Streptococcus mitis and Streptococcus sanguis: an in vitro study. J Contemp Dent Pract. 2012; 13(6):769-72. PMID: 23404001.
21. Alzohairy MA. Therapeutics role of Azadirachta indica (Neem) and their active constituents in diseases prevention and treatment. Evid Based Complement Alternat Med. 2016; 2016: 7382506. PMID: 27034694.
22. Tiwari V, Darmani NA, Yue BY, Shukla D. In vitro antiviral activity of neem (Azardirachta indica L.) bark extract against herpes simplex virus type-1 infection. Phytother Res. 2010; 24(8):1132-40. PMID: 20041417.
23. Badam L, Joshi SP, Bedekar SS. 'In vitro' antiviral activity of neem (Azadirachta indica. A. Juss) leaf extract against group B coxsackieviruses. J Commun Dis. 1999; 31(2): 79-90. PMID: 10810594.
24. Arora R, Chawla R, Marwah R, Arora P, Sharma RK, Kaushik V, Goel R, Kaur A, Silambarasan M, Tripathi RP, Bhardwaj JR. Potential of complementary and alternative medicine in preventive management of novel H1N1 Flu (Swine Flu) pandemic: thwarting potential disasters in the bud. Evid Based Complement Alternat Med. 2011; 2011: 586506. PMID: 20976081.
25. Siddiqui BS, Ali SK, Ali ST, Naqvi SN, Tariq RM. Variation of major limonoids in Azadirachta indica fruits at different ripening stages and toxicity against Aedes aegypti. Nat Prod Commun. 2009; 4(4):473-6. PMID: 19475987.
26. Marsh S. Doctors, nurses, porters, volunteers: the UK health workers who have died from Covid-19. Available from: https://www.theguardian.com/world/2020/apr/16/doctors-nurses-porters-volunteers-the-uk-health-workers-who-have-died-from-covid-19[Accessed 5 May, 2020].

Annexure: 1 (Operational Definitions)

1. COVID-19: An RNA based virus, member of coronavirus family responsible for the current pandemic that has affected nearly 3.7 million cases worldwide. The symptoms are similar to the infection of caused by SARS (severe acute respiratory syndrome). We intend to induct PCR confirmed COVID-19 cases which are within 7 days on infection onset.
2. PCR: Polymerase chain reaction. It will be used to amplify genetic material required for testing. Both the baseline and endpoint specimens will be obtained from posterior pharyngeal wall by trained personnel using proper PPE.
3. Cytokine array: A multiplex ELISA method will be used to detect fluorescence antibody against predefined antigen. We will study the following cytokine profile IL-2, IL-4, IL-6, IL-10, TNF-α, IFN-γ and IL-17 among COVID-19 patients in the present study.
4. Neem extract: Biological name *Azardirachta indica.* This has a number of active chemicals that have antiviral activity. A solution will be compounded at University Karachi, Chemistry department laboratory to be used as oral rinse and nasal lavage for COVID-19 patients.
5. PPE: This refers to Personal protective equipment including protective clothing, N-95 mask, eye wear and other garments or equipment designed to protect the person or infection.
6. Gargle: Transiently keeping some fluid in the oral cavity/ oro-pharynx followed by spitting off with an objective of physically rinsing off the mucosal membrane. We will use five different solutions as gargles.
7. Nasal lavage: Passing a fluid in the nose following by sneezing like action to mechanically clear the nasal cavity. The gargling solutions will employed in nasal lavage using a special syringe shown below.

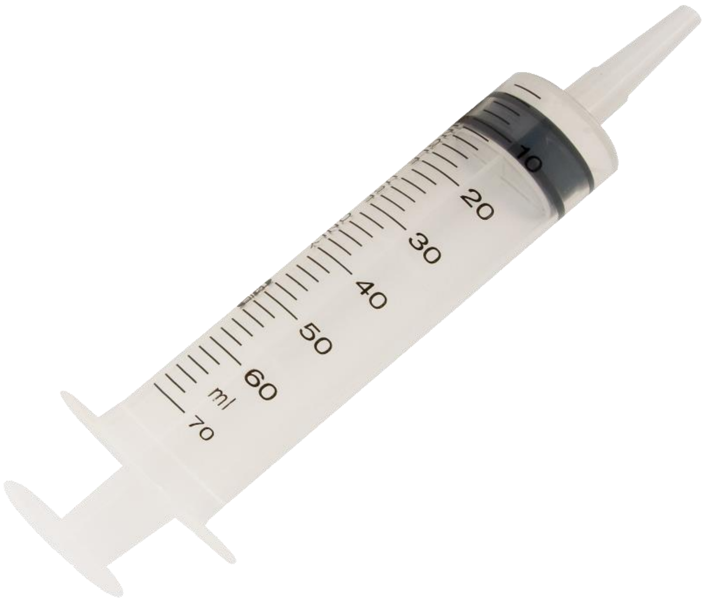

Figure 2: Syringe to be used for nasal lavage
